# The weaponization of medical referrals and evacuations during the genocide in Gaza: a brief report and call to action

**DOI:** 10.1186/s42506-026-00214-5

**Published:** 2026-03-19

**Authors:** Anas Ismail, Muhammed Abu Salmiya, Motasem Salah, Craig Jones

**Affiliations:** 1 Population Health Sciences Institute, Newcastle University, Newcastle Upon Tyne, UK; 2 School of Geography, Politics and Sociology, Newcastle University, Newcastle Upon Tyne, UK; 3 Al Shifa Hospital, Gaza, Palestine; 4 University College of Applied Science, Gaza, Palestine

## Abstract

**Background:**

The Palestinian healthcare system has historically relied on referrals of patients to health facilities across Palestine’s borders. Patients referred typically suffered from chronic conditions or congenital anomalies for which the governmental health facilities in Palestine lacked treatment or diagnostic options. The ongoing Israeli genocide on the Palestinian population has destroyed much of the health system and facilities in Gaza and caused tens of thousands of traumatic injuries that need referral abroad, on top of the cohort of patients with chronic conditions and congenital anomalies.

**Findings:**

Statistics published by the WHO-oPt show that 7,841 patients have been allowed medical evacuation and referral from Gaza between the start of the war in October 2023 and 29 September 2025. About two-thirds of all patients (*n* = 5,000. 63.8%) were medically evacuated before the Israeli military forces occupied the Rafah border crossing with Egypt on 7 May 2024, and 1,702 (21.7%) patients were evacuated between 19 January 2025 and 17 March 2025 as part of the ceasefire agreement. Egypt has received the majority of patients (*n* = 3,995, 51%) who have been medically evacuated. Excluding the two periods mentioned, a clear Israeli policy emerged since occupying the Rafah border crossing, which weaponized healthcare by preventing patients from Gaza from being medically evacuated to travel abroad to receive life-saving healthcare.

**Conclusions:**

The Israeli policy of weaponizing the referrals and medical evacuations has resulted in excess mortality that merits further research and quantification, as patients succumbed to their medical conditions or injuries while waiting to exit Gaza. Action needs to be taken by host countries to step up their efforts to receive more patients from Gaza and put more pressure on Israel to facilitate the safe evacuation process of these patients.

## An ongoing genocide

Since October 2023, the people of Gaza have been facing an ongoing genocide [1–3] that has wrought devastation on all aspects of life, including the near-complete destruction of the health system. The Palestinian Ministry of Health in Gaza (MOH) reported 65,502 people killed and 167,376 people injured directly from military actions as of 25 September 2025, and of them 12,939 killed and 55,335 injured since Israel’s resumption of the fighting on 18 March 2025 (MOH social media posts). Studies published in The Lancet report that the MOH numbers, while reliable, are underestimates. The actual number of those killed could be up to 41% higher than reported [4, 5]. Furthermore, similar to other conflicts around the world [6] hospitals and health facilities in Gaza have been systematically destroyed, forcing over half of the hospitals to close entirely, with the rest only functioning partially [7, 8].

Outside medical referrals (OMRs) have historically been an integral part of the healthcare system for Palestinians. The destinations of OMRs during the 1950s and 1960s were mostly to Egypt and Jordan, which had control over the Gaza Strip and the West Bank, respectively. After the 1967 *Naksa* and the Israeli military occupation of the rest of Palestine, Palestinian patients were regularly referred to Israeli hospitals, once limited treatment options were exhausted in local hospitals [9, 10]. This dependence on OMRs continued. Between 2019 and 2021, there were 284, 601 referrals issued for Palestinian patients, and 27% of them (around 77,000 referrals) were for patients from Gaza [11]. The large number of referrals means that OMRs constitute the largest source of health expenditure in the MOH budget; for example, the MOH in Ramallah reported that in 2022, OMRs cost about 1.1 billion New Israeli Shekel (NIS), constituting 41.2% of all health expenditure [12].

These regular referrals are mostly for patients with chronic and life-threatening conditions, such as cancer and congenital malformations. In the ongoing genocide, dependence on OMRs has deepened. The unprecedented numbers of often very complex war injuries, combined with the destruction of health facilities on top of the chronic shortages in medicines and supplies from the long-standing military blockade on Gaza [13], necessitated medical evacuation of injured and sick patients to be treated abroad. The influx of trauma patients in Gaza significantly exacerbates the pre-existing volume of routine medical referrals. Abiding by principles of the Geneva Conventions, which require the sick and injured “not willfully be left without medical assistance and care” and of evacuation of “wounded, sick, infirm… cases” [14], would have meant exponentially scaling up the referral process to attend to the needs of thousands of Palestinian injured and sick patients.

Instead, Israeli authorities sought a policy of weaponizing healthcare – defined by Fouad et al. [15] as “a strategy of using people’s need for health care as a weapon against them by violently depriving them of it”. Such weaponization can be in the form of attacking and destroying health facilities, killing and abducting healthcare workers, or preventing the passage of ambulances and other medical vehicles. All of these forms occurred in Gaza as Israeli forces weaponized healthcare by systematically targeting healthcare facilities in all parts of the Gaza Strip, leaving more than half of the hospitals non-functional and the remaining ones only partially functional [7] and by killing over 1,200 healthcare workers and detaining over 380 others, many of whom are specialists whose expertise is extremely hard to replace [16]. In this report, our focus is on Israel’s policy of depriving the injured and the sick of the much-needed medical evacuations.

### Tracking medical evacuations

The WHO-oPt tracks the numbers of patients who need medical evacuations and those who were able to travel and be medically evacuated as well as their country destinations. This can be found on their webpage [17]. The webpage lists a number of infographics that track the number of medical evacuations from October 2023 until the time of writing (September 2025). Additionally, a medical evacuations dashboard [18] shows the number of medical evacuations from July 2024 until the time of writing. These numbers come mostly from the MOH, which initiates the medical referral process for patients after they are evaluated by physicians and a medical referrals committee. The MOH itself has an internal tracking system, Sehatty (https://www.sehatty.ps/public/moh-citizen#), which allows the MOH officials to manage and patients to track medical evacuations and their status, though the MOH has not published statistics from this tracking system.

## Weaponization of medical evacuations

Studying the reports issued by the WHO-oPt reveals a clear Israeli policy of weaponizing the medical evacuations, particularly after the Israeli military forces took over the Rafah border crossing with Egypt on 7 May 2024. Table 1 shows the number of evacuated patients in each period in relation to the occupation of the Rafah border and the January 2025 ceasefire, based on reporting by WHO-oPt [19]. We can clearly see that patients were generally allowed evacuation in two periods: before the occupation of the Rafah border crossing between 7 October 2023 and 6 May 2024 (5,000 patients) and as part of the January 2025 ceasefire between 19 January 2025 and 17 March 2025 (1,702 patients). On the other hand, Israel’s weaponization of the medical evacuations can be clearly seen in the significant drop of numbers of patients evacuated in the other two periods: after Israel’s occupation of the Rafah border crossing (459 patients) and after Israel resumed the fighting on 18 March 2025 and up to 29 September 2025 (680 patients).

**Table 1:** The number of evacuations in each of the periods of the fighting between 7/10/2023 and 29/9/2025 as reported by WHO-oPt

Period	Events	Number of evacuated patients (%)
7/10/2023–6/5/2024	Before the occupation of the Rafah border crossing	5,000 (63.77%)
7/5/2024–18/1/2025	Between the occupation of the Rafah border crossing and the January 2025 ceasefire.	459 (5.85%)
19/1/2025–17/3/2025	The January 2025 ceasefire	1,702 (21.71%)
18/3/2025–29/9/2025	After Israel resumes the fighting	680 (8.67%)
Total		7,841

The number of patients still requiring referral and medical evacuation is estimated to be over 15,000 patients, both suffering medical conditions and traumatic injuries from the Israeli attacks, and, the number of patients allowed medical evacuation is only a minority of patients in need of evacuation. For example, in its latest available annual report in 2022, the MOH reported 2,047 new cases of cancer in 2022 and 7,569 new cases since 2018 [20]. By extrapolating these numbers, we can assume that there were around 6,000 new cancer cases in Gaza in 2023, 2024, and 2025. All of these patients would require referrals abroad for diagnostic and therapeutic procedures that are not available in Gaza now. However, only 716 oncology patients were evacuated until 29 September 2025, resulting in excess mortality among patients who were not evacuated. The same can be said about other types of patients who were regularly referred abroad, such as, among others, those with medical conditions (cardiovascular, congenital, neurosurgical conditions, etc.), as well as traumatic injuries sustained in the two years of genocide.

The significant differences in the numbers of patients evacuated in the different periods speak to the potential capacity to evacuate patients that can and should be fully utilized. Furthermore, the overall number of patients who were allowed medical evacuation is nowhere near the potential capacity for the referral and medical evacuation mechanisms, given that, for example, nearly 1,500 requests were approved in July 2023 alone for patients to be referred out of Gaza [21].

Such weaponization of healthcare through the restrictions on referrals and medical evacuations is not new and has been a routine practice by Israeli authorities, which denied or delayed ‘security approvals’ needed by patients and their companions, particularly from Gaza, to cross the border to reach their destination hospitals. Between 2019 and 2021, 35% of the ‘security’ approval applications for Gaza patients were denied or delayed, and patients could not attend their appointments in the receiving hospitals [11]. In July 2023, for example, 1 out of 5 patients and over half of the patient companions (51%) had their ‘security permits’ denied or delayed [21]. Such weaponization of healthcare is known to have caused increased mortality and morbidity among patients whose ‘security permits’ were denied or delayed [22–24]. Moreover, companions whose permits are denied can sometimes be parents to children who need to be referred to receive life-saving or life-altering treatment. This resulted, at some points in time, in as high as 40% of sick children (numbering in the thousands) being referred to receive medical care without a parent accompanying them [25], leading to complex ethical dilemmas and unnecessary psychological suffering for the children and their parents.

## A call to action

An unknown number of injured and sick patients have died as a result of the weaponization of healthcare and the prevention of medical evacuations in the war. Also, an unknown number of patients will suffer preventable long-term sequelae because their injuries or sicknesses weren’t treated in a timely manner as a result of the severe restrictions on medical evacuations.

Small groups of patients (fewer than 50 per day) were medically evacuated during the brief ceasefire between 19 January 2025 and 17 March 2025 (daily evacuations started on 1 February 2025) as part of the ceasefire agreement, and 680 patients were allowed evacuation after the ceasefire broke. This is a further testament to Israel’s weaponization of healthcare and its use as part of its military campaign in Gaza. Yet, these numbers are a very small portion of those who need to be urgently evacuated, particularly in light of the resumption of the war and increasing numbers of injured.

More injured and sick patients will continue to die or suffer preventable complications unless the capacity to medically evacuate them is exponentially increased and Israel assumes its responsibility for the health of the people it occupies, as affirmed in the 1949 Geneva Conventions and 1977 Additional Protocols. In parallel, countries need to step up their efforts to accept and receive the injured and sick patients from Gaza to save as many lives as possible. Finally, international organizations, including UN Agencies and the International Red Cross Committee, should continue to facilitate the entry of much-needed medical equipment and foreign missions as well as advocate for and be present on the ground to protect what remains of the Palestinian health system in Gaza, particularly hospitals. This may reduce the suffering of patients who need to be referred while they are stranded in Gaza.

Palestinian healthcare workers and emergency medical teams in Gaza continue to do their best in light of the circumstances to save the lives of their patients, but unless these actions are taken, the death toll from the genocide will continue to increase even after the fighting stops.

## Limitations

This brief report aimed to highlight the urgent and grim reality of patients in Gaza in the midst of the ongoing genocide. The report is limited in its reporting as it does not rely on primary data collected on medical evacuations and, instead, makes use of numbers and figures in published reports and infographics. Second, we did not present mortality data on patients who have died while waiting for their evacuations, as this was not available to us. This can be an area for further research in the future.

## Data Availability

Not applicable.
